# Cytokine profiles of umbilical cord blood mononuclear cells upon *in vitro* stimulation with lipopolysaccharides of different vaginal gram-negative bacteria

**DOI:** 10.1371/journal.pone.0222465

**Published:** 2019-09-19

**Authors:** Edith Reuschel, Martina Toelge, Kathrin Entleutner, Ludwig Deml, Birgit Seelbach-Goebel

**Affiliations:** 1 Department of Obstetrics and Gynecology, University of Regensburg, Hospital of the Barmherzige Brueder, Clinic St Hedwig, Regensburg, Germany; 2 Institute of Medical Microbiology, University Hospital Regensburg, Regensburg, Germany; University of St Andrews, UNITED KINGDOM

## Abstract

Inflammatory immune responses induced by lipopolysaccharides (LPS) of gram-negative bacteria play an important role in the pathogenesis of preterm labor and delivery, and in neonatal disorders. To better characterize LPS-induced inflammatory response, we determined the cytokine profile of umbilical cord blood mononuclear cells (UBMC) stimulated with LPS of seven vaginal gram-negative bacteria commonly found in pregnant women with preterm labor and preterm rupture of membrane. UBMC from ten newborns of healthy volunteer mothers were stimulated with purified LPS of *Escherichia coli*, *Enterobacter aerogenes*, *Klebsiella pneumoniae*, *Proteus mirabilis*, *Acinetobacter calcoaceticus*, *Citrobacter freundii*, and *Pseudomonas aeruginosa*. UBMC supernatants were tested for the presence of secreted pro-inflammatory cytokines (IL-6, IL-1β, TNF), anti-inflammatory cytokine (IL-10), TH_1_-type cytokines (IL-12, IFN-γ), and chemokines (IL-8, MIP-1α, MIP-1β, MCP-1) by Luminex technology. The ten cytokines were differentially induced by the LPS variants. LPS of *E*. *coli* and *E*. *aerogenes* showed the strongest stimulatory activity and *P*. *aeruginosa* the lowest. Interestingly, the ability of UBMC to respond to LPS varied greatly among donors, suggesting a strong individual heterogeneity in LPS-triggered inflammatory response.

## Introduction

Preterm birth is the most common cause of neonatal death [1]. It is associated with increased neonatal short- and long-term morbidity including severe damages to the brain, bowel, lungs, eyes and developmental defects [2–5]. Preterm labor (PTL) is defined as labor arising from premature uterine contractility and occurs prior to 37 weeks of gestation in human. In the United States, approximately 10% of all births are diagnosed as preterm [6] and 15 million premature babies are estimated to be born annually worldwide as a result of PTL [7].

As many as 40% of spontaneous preterm births may be attributed to antenatal infection [8] and nearly half of the three million neonatal deaths that occur annually are due to bacterial infections [9]. Ascending genital tract infections of the vagina represent a major cause of PTL, spontaneous preterm birth and preterm rupture of membrane (PROM). Intra-amniotic infection has been causally linked to spontaneous PTL [10]. Indeed, preterm deliveries ensue frequently from an increase in bacterial colonization, which originates as bacterial vaginosis and spreads over gestational tissues including choriodecidua, fetal membranes, amniotic cavity, and eventually the fetus. Bacterial vaginosis up to the 26th week of gestation increases a woman´s risk of preterm delivery by 50% [11], and intrauterine infection is often associated with chorioamnionitis [12,13]. Neonates born to mothers with clinical chorioamnionitis are at risk for sepsis [14–16], meconium aspiration syndrome [17], neonatal encephalopathy [18], long-term neurodevelopmental disabilities including cognitive impairment [19,20] and cerebral palsy [21].

PTL exhibit a common feature whereby an increase in inflammatory mediators, such as TNF, IL-1, IL-6 and IL-8, is observed in the amniotic fluid before the onset of uterine contraction. Thus, it is currently assumed that PTL is triggered by premature emergence of these pro-inflammatory mediators in gestational tissues [22]. These pro-inflammatory cytokines might also be the link between prenatal intrauterine infection and intraventricular hemorrhage, resulting in neonatal white matter damage and subsequent cerebral palsy in preterm delivered newborns [23,24]. Maternal systemic inflammation during critical windows of pregnancy may even predispose the neonate to autism spectrum disorders [25] and schizophrenia [26].

Gram-negative bacteria are known to cause severe neonatal infections and associated complications, such as meningitis, cerebral abscesses, septicaemias and sudden unexpected death in neonates and infants [27–32]. Gram-negative bacteria express a glycolipid component called endotoxin or lipopolysaccharide (LPS) which participates in the physiological functions of the outer bacterial membrane and is the principal inflammatory component of the gram-negative bacterial envelope [33,34]. LPS signals through Toll-like receptor 4 (TLR4), a member of the TLR family belonging to the pattern recognition receptor family. LPS stimulation via TLR4 results in inflammatory cytokine production, which is responsible for activating the innate immune system [35]. Various cytokines are produced in PTL caused by bacterial infections, via the LPS/TLR4 pathway, causing maternal but also fetal pathologies, such as brain damage, pulmonary or intestinal complications [36–38]. LPS-mediated inflammatory response is clearly correlated to prematurity or fetal loss [39–42]. A role of the CX3CL1-CX3CR1 chemokine signaling pathway through intrauterine recruitment of macrophages and the enhancement of macrophage-derived inflammatory mediators was recently demonstrated in LPS-induced PTL in mice [43].

LPS are large molecules consisting of a lipid (Lipid A) covalently bound to a polysaccharide [33,34]. Because of the limited variability in their structure, it was initially assumed that all LPS molecules have comparable biological activities. However, it has now been shown that LPS from different bacterial species are structurally and functionally distinct. *In vitro* experiments have demonstrated great variance in the capacity of different LPS species to induce the synthesis of cytokines [44]. The shape of the Lipid A determines the bioactivity of the LPS [45]. Lipid A that adopts a conical shape conformation (e.g. *E*. *coli*) is more active than one that adopts a cylindrical conformation [46]. Gram-negative bacteria have developed numerous mechanisms for the modification of lipid A. These mechanisms include the expression of enzymes adding or removing acyl chains phosphate groups, or altering the acylation state of their LPS in response to environmental changes, or catalyzing the addition of chemical groups into the phosphates [47]. These modifications affect the recognition of LPS by TLR4 and thus impact the downstream inflammatory and immune responses [48]. Differences in TLR4 function and regulation in adult and neonatal monocytes have also been described [49–51]. These may account for the attenuated cytokine responses in neonates and the immaturity of infection control noted in preterm and term newborns in association with impaired TLR signaling [52].

We previously examined the distribution of gram-negative bacteria in vaginal and cervical swabs of 239 pregnant women with PROM or PTL at our clinic and identified seven species at the following frequency: *E*. *coli* (90%), Klebsiella spp. (12%), Proteus spp. (9%), Pseudomonas spp. (5%), *Morganella morganii* (2%), Enterobacter spp. (2%), Citrobacter spp. (2%) and Acinetobacter (2%) [53].

We hypothesized that LPS of different gram-negative bacteria present in the vagina of pregnant women induce the release of different cytokine and chemokine profiles, possibly resulting in distinct immunomodulatory activities. We verified this hypothesis by using an *in vitro* system of freshly isolated umbilical cord blood mononuclear cells (UBMC) stimulated with purified LPS of clinically relevant gram-negative vaginal bacteria. UBMC isolated from ten uncomplicated at-term deliveries were stimulated with purified LPS and the release of a broad panel of immunomodulatory cytokines and chemokines was measured using the Luminex technology.

## Materials and methods

### Umbilical cord blood source

Informed consent was obtained from ten voluntary healthy mothers. Participants were non-smokers, were not under medical treatment, showed no signs of infection during pregnancy and delivered at term. Umbilical cord blood was collected immediately at birth in citrate bags, following ligation of the umbilical cord, and processed within 12 hours. The protocol of the study was conducted in accordance with the World Medical Association Declaration of Helsinki and approved by the Ethical Commission of the University of Regensburg, Center for Clinical Studies (Approval number 06/098).

### Isolation of umbilical cord blood mononuclear cells (UBMC)

The freshly citrated umbilical cord blood was diluted 1:3 with PBS, and 25 to 30 ml diluted blood was carefully laid over 15 ml Pancoll (Lymphocyte sep. medium/ Pancoll human, density 1.077 g/ml, PAN, Biotech, Aidenbach, Germany). UBMC were separated by density gradient centrifugation (800 x g, 30 min) at room temperature (RT), harvested as a single interface layer, and washed three times with 45 ml PBS. Contaminating erythroblasts were eliminated by lysis in 1 ml erythrocyte lysis buffer (BD Pharm Lyse^TM^ LYSING BUFFER, Cat. No. 555899) for 10 min at RT, followed by two additional washes in 45 ml PBS. Cell pellets of each donor were resuspended in T-cell medium (RPMI 1640 medium, Gibco, Paisley, Scotland) supplemented with 10% heat-inactivated human AB serum, and enumerated using a Neubauer counting chamber. Total cell number was adjusted to 2 x 10^5^ cells per 90 μl in T-cell medium.

### Purified LPS

Purified LPS of selected gram-negative bacteria species were kindly provided by the reference center Research Center Borstel, Leibniz Lung Center (Germany). LPS of the following gram-negative bacteria species were extracted from isolates of various origins as described: *E*. *coli* GH58 [54], *E*. *aerogenes* 1033 [55], *P*. *mirabilis* rough mutant R45 [56], *K*. *pneumoniae* rough mutant R20/01 [57], *A*. *calcoaceticus* NCTC 10305 [58], *C*. *freundii* (S-Form LPS purified by gel permeation chromatography) [59], and *P*. *aeruginosa* PAO1 [60].

### Stimulation of UBMC with LPS

Freshly isolated UBMC (2 x 10^5^) were seeded in triplicate (90 μl per well) into polypropylene plastic 96-well plates (Nunc, Roskilde, Denmark). Unless otherwise indicated, cells were stimulated with 37.5 ng/ml purified LPS in 100 μl final volume for 36 hours at 37°C in a humidified atmosphere containing 5% CO_2_. UBMC stimulated with PBS served as a negative control. The synthetic bacterial lipopeptide and TLR1/2 agonist PAM3CSK4 [61] (L2000, EMC Microcollections, Tuebingen) was tested in parallel at the concentration of 37.5 ng/ml. Cell-free supernatants of stimulated cells were harvested by low speed centrifugation (300 x g, 10 min) and stored at -80°C.

### Determination of cytokine and chemokine concentrations in UBMC supernatants

The levels of cytokines and chemokines in the UBMC supernatants were determined applying the Luminex technology (MicroBIOMix GmbH, Regensburg). A pilot experiment on one subject was performed using the Human Cytokine 30-Plex panel (LHC6003, Invitrogen/ThermoFisher), which includes the following cytokines, chemokines and growth factors: IL-1β, IL-1-Receptor Antagonist (IL-1RA), IL-2, IL-2 receptor (IL-2R), IL-4, IL-5, IL-6, IL-7, IL-8, IL-10, IL-12 (protein 40/protein 70), IL-13, IL-15, IL-17, TNF, interferons IFN-α and IFN-γ, granulocyte-macrophage colony stimulating factor (GM-CSF), macrophage inflammatory proteins MIP-1α and MIP-1β, interferon-γ-induced protein 10 (IP-10), monokine induced by interferon-γ (MIG), eotaxin, regulated and normal T-cell expressed and secreted (RANTES), monocyte chemoattractant protein 1 (MCP-1), vascular endothelial growth factor (VEGF), granulocyte colony stimulating factor (G-CSF), epidermal growth factor (EGF), fibroblast growth factor (FGF) and human growth factor (HGF). Further Luminex assays were conducted using the following human singleplex beads kits (Invitrogen/ThermoFisher): IL-1β (LHC0011), IL-10 (LHC0101), IL-6 (LHC0061), IL-8 (LHC0081), IL-12 [p40/p70] (LHC0121), TNF (LHC3011), IFN-γ (LHC4031), MIP-1α (LHC1021), MIP-1β (LHC1051), and MCP-1 (LHC1011). Experiments were performed using the Human Extracellular Protein Buffer Reagent Kit (LHB0001, Invitrogen/ThermoFisher) according to the manufacturers' protocol. The assays were performed in 96-well filter bottom plates. The beads were protected from light throughout the procedure. The lyophilized standard was reconstituted in 2 ml assay diluents and 1:3 serial dilutions were undertaken to generate a seven standard concentration set, while diluent alone served as blank. The filtered plates were pre-washed with 200 μl of wash solution/well for 30 seconds. Wash solutions were aspirated using a vacuum manifold and the bottom of the plate was blotted on paper towels to remove residual liquid. The concentrated bead mix was diluted 1:20 in wash solution. The bead solution was vortexed and sonicated immediately prior to adding 50 μl/well. The plate was washed twice with 200 μl wash solution/well as above. Incubation buffer (50 μl) and each standard (100 μl) were added in duplicate. Assay diluent (50 μl) was added to each well followed by the addition of the sample (50 μl). The plate was covered and incubated for 2 h at RT on an orbital plate shaker (600 rpm). Afterwards, the liquid was removed using a vacuum manifold, and the plate was washed twice. A biotinylated detection antibody (100 μl) was added and plate was covered and incubated on the shaker for 1 h at RT. The plate was washed twice prior to addition of Streptavidin-RPE (100 μl), then covered and incubated for 30 min at RT on the shaker. Finally, the plate was washed three times. Wash solution (110 μl) was added to each well, the plate was covered, placed on the orbital shaker and incubated for 2–3 min at RT prior to analysis. Mean fluorescence intensity (MFI) was acquired using the Luminex xMAP 100 system (Luminex Corp.). Software was set to acquire data using 70 μl sample and count 100 events per single bead set.

### Statistical analyses

Cyto- and chemokine-concentrations were calculated using a 4- or 5-parameter logistic fit curve generated from the 7 standards using the Liquichip Analyzer software (Qiagen, Germany) and expressed in ng/ml in accordance with International Standards. Statistical differences between measured values in stimulated and unstimulated conditions were analyzed using a Mann-Whitney *U*-test. *P* values less than 0.05 were considered statistically significant. Statistical tests were performed using SPSS 15 (for Windows).

## Results

Purified LPS from seven gram-negative vaginally occurring bacteria commonly found in pregnant women with PTL and PROM, namely *E*. *coli* GH58, *E*. *aerogenes* 1033, *P*. *mirabilis* rough mutant R4, *K*. *pneumoniae* rough mutant R20/01, *A*. *calcoaceticus* NCTC 10305, *C*. *freundii* (S-Form), and *P*. *aeruginosa* PAO1 [53], were used to stimulate UBMC freshly isolated at birth from ten healthy at-term neonates. Secreted cytokines were quantified in the cell culture supernatant of stimulated UBMC using the Luminex technology. The characteristics of the mothers and respective newborns are shown in Table 1. Median (range) time from blood collection to processing was 7.0 (2.5–12.0) hours.

**Table 1:** Characteristics of the 10 mothers and respective neonates, as well as storage time of umbilical cord blood from collection at delivery until isolation of UBMC^a^.

Subject number	Maternal age (years)	Parity	Gestational age (weeks + days)	Mother´s BMI^a^ before pregnancy	Baby’s weight at birth (g)	Baby’s sex	Time to UCB^a^ processing (hours)
1	22	1	40 + 3	22.9	3865	Male	11
2	35	1	38 + 6	24.3	3060	Male	3
3	25	2	37 + 0	18.6	3045	Female	12
4	33	2	40 + 1	21.6	3920	Female	9
5	37	3	40 + 4	19.8	3610	Female	9
6	30	2	41 + 2	20.9	3740	Female	5
7	33	2	40 + 0	23.5	3220	Female	3.5
8	27	4	42 + 1	17.5	3850	Male	2.5
9	33	1	40 + 2	26.8	3360	Female	10
10	24	1	39 + 1	25.8	3125	Male	5
**Median**	**31.5**	**2**	**40 + 1.5**	**22.25**	**3485**		**7.0**
**Range**	**22–37**	**1–4**	**37–42 + 0–6**	**17.5–26.8**	**3045–3920**		**2.5–12.0**

^a^Abbreviations: BMI, body mass index; UCB, umbilical cord blood; UBMC, umbilical cord blood mononuclear cells.

**Fig 1:**
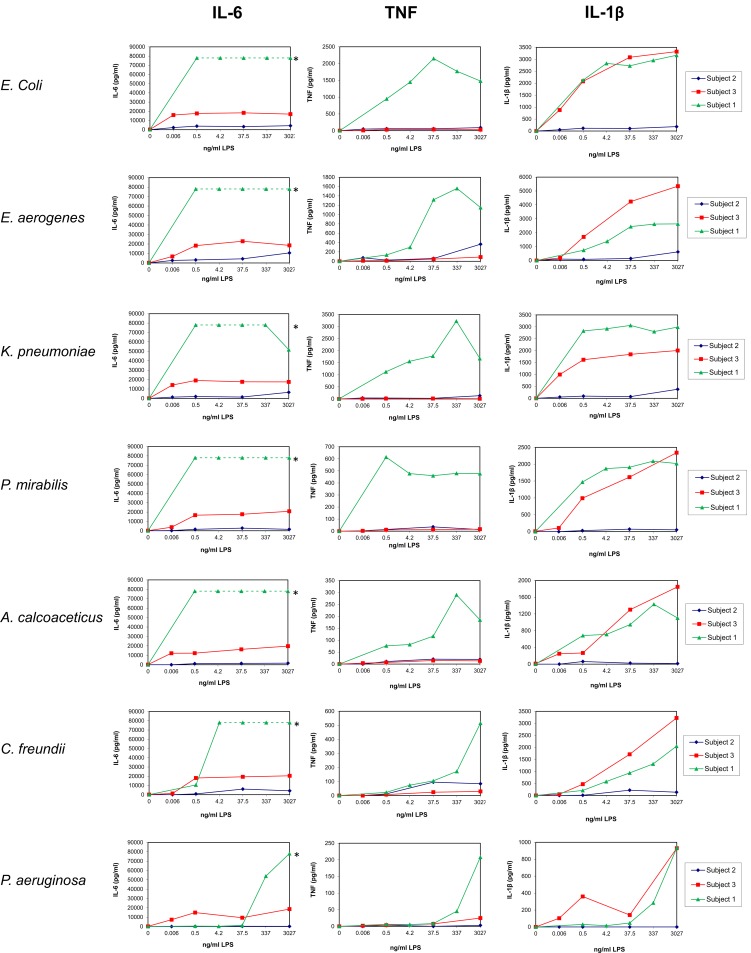
Dose response of LPS on the release of pro-inflammatory cytokines by UBMC. UBMC isolated from three of the ten deliveries (Subjects 1–3; Table 1) were stimulated for 36 hours with increasing amounts (0–3027 ng/ml) of purified LPS derived from the indicated seven gram-negative bacteria (*E*. *coli*, *E*. *aerogenes*, *K*. *pneumoniae*, *P*. *mirabilis*, *A*. *calcoaceticus*, *C*. *freundii*, *P*. *aeruginosa*). The levels of pro-inflammatory cytokines (IL-6, IL-1β, TNF) released in the supernatants of UBMC were measured by Luminex, as described in the Methods section. UBMC of Subject 1 released levels of IL-6 above the detection range of the Luminex assay (saturated signal), as indicated by the green dotted line and the (*) sign.

**Fig 2:**
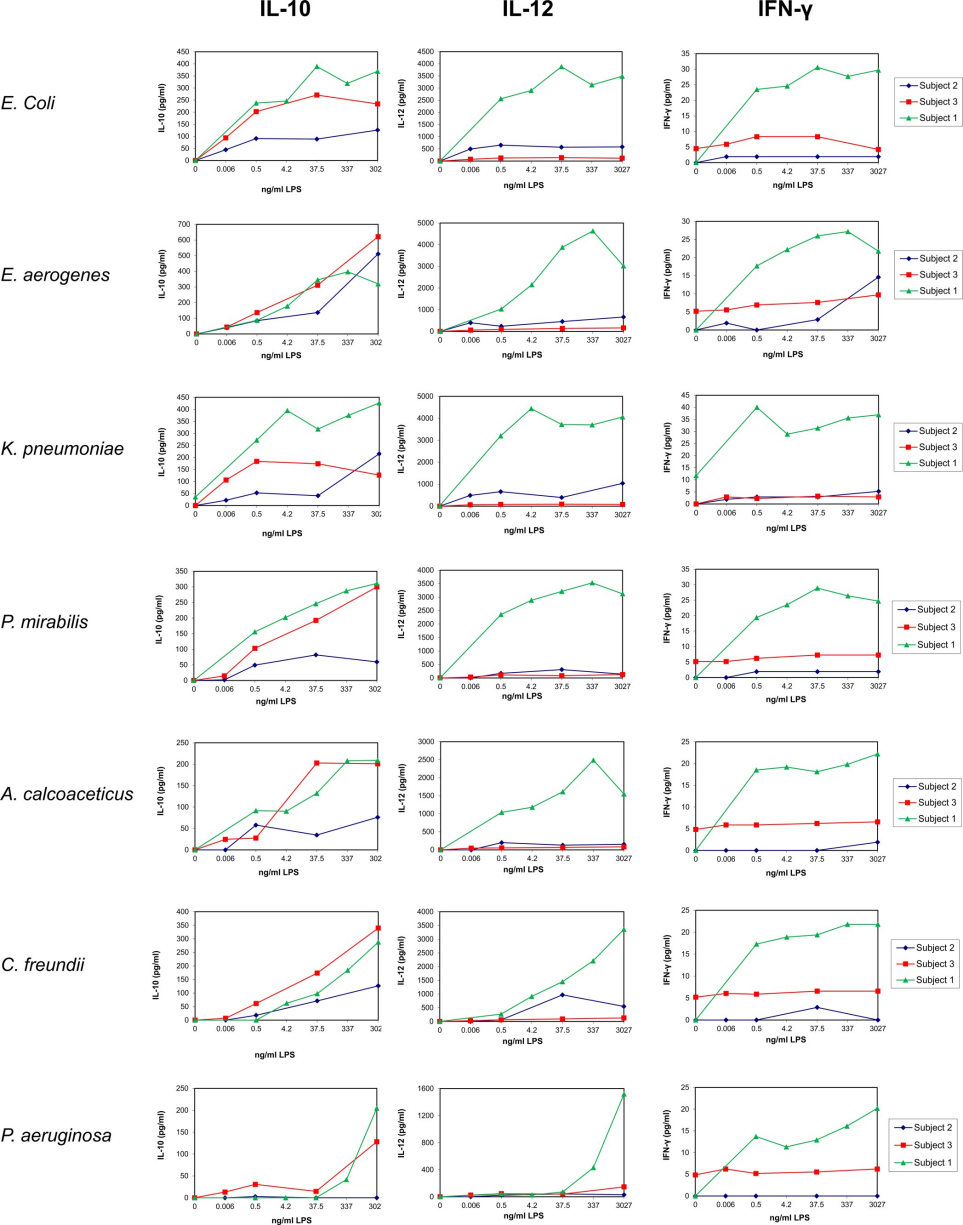
Dose response of LPS on the release of anti-inflammatory and TH1-type cytokines by UBMC. UBMC isolated from three of the ten deliveries (Subjects 1–3; Table 1) were stimulated for 36 hours with increasing amounts (0–3027 ng/ml) of purified LPS derived from the indicated seven gram-negative bacteria (*E*. *coli*, *E*. *aerogenes*, *K*. *pneumoniae*, *P*. *mirabilis*, *A*. *calcoaceticus*, *C*. *freundii*, *P*. *aeruginosa*). The levels of anti-inflammatory cytokine (IL-10) and TH_1_-type cytokines (IL-12, IFN-γ) released in the supernatants of UBMC were measured by Luminex, as described in the Methods section.

**Fig 3:**
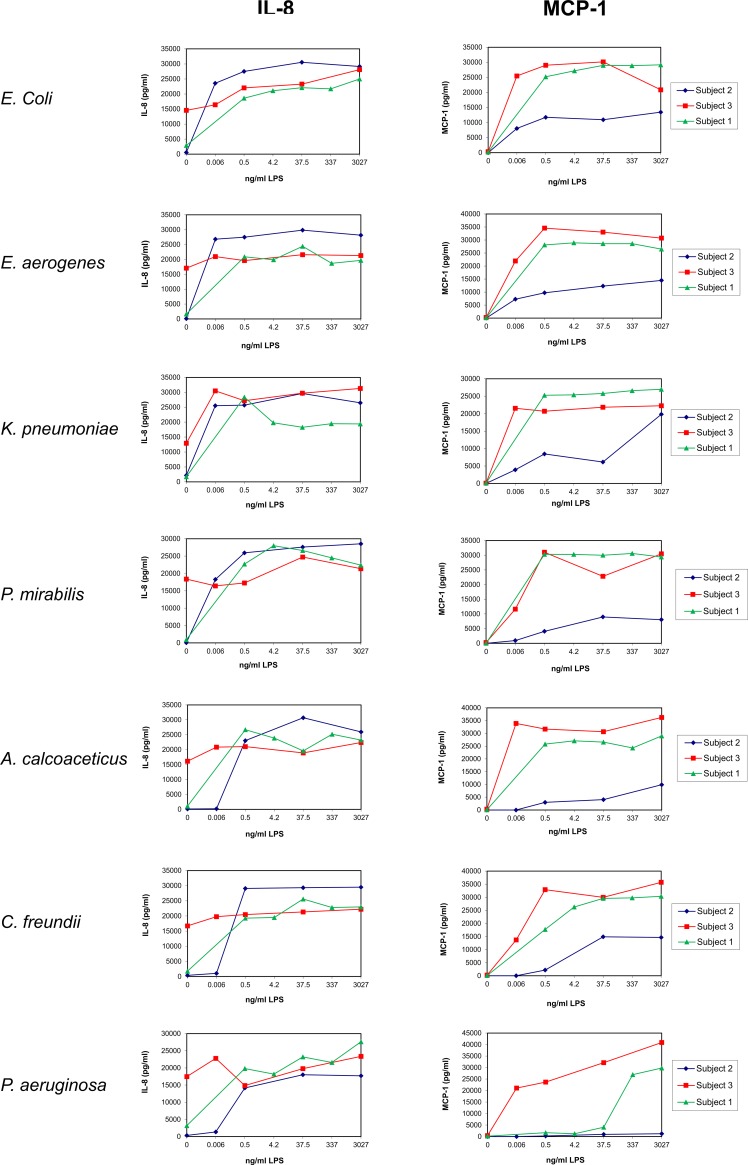
Dose response of LPS on the release of chemokines by UBMC. UBMC isolated from three of the ten deliveries (Subjects 1–3; Table 1) were stimulated for 36 hours with increasing amounts (0–3027 ng/ml) of purified LPS derived from the indicated seven gram-negative bacteria (*E*. *coli*, *E*. *aerogenes*, *K*. *pneumoniae*, *P*. *mirabilis*, *A*. *calcoaceticus*, *C*. *freundii*, *P*. *aeruginosa*). The levels of chemokines (IL-8, MCP-1) released in the supernatants of UBMC were measured by Luminex, as described in the Methods section.

**Fig 4:**
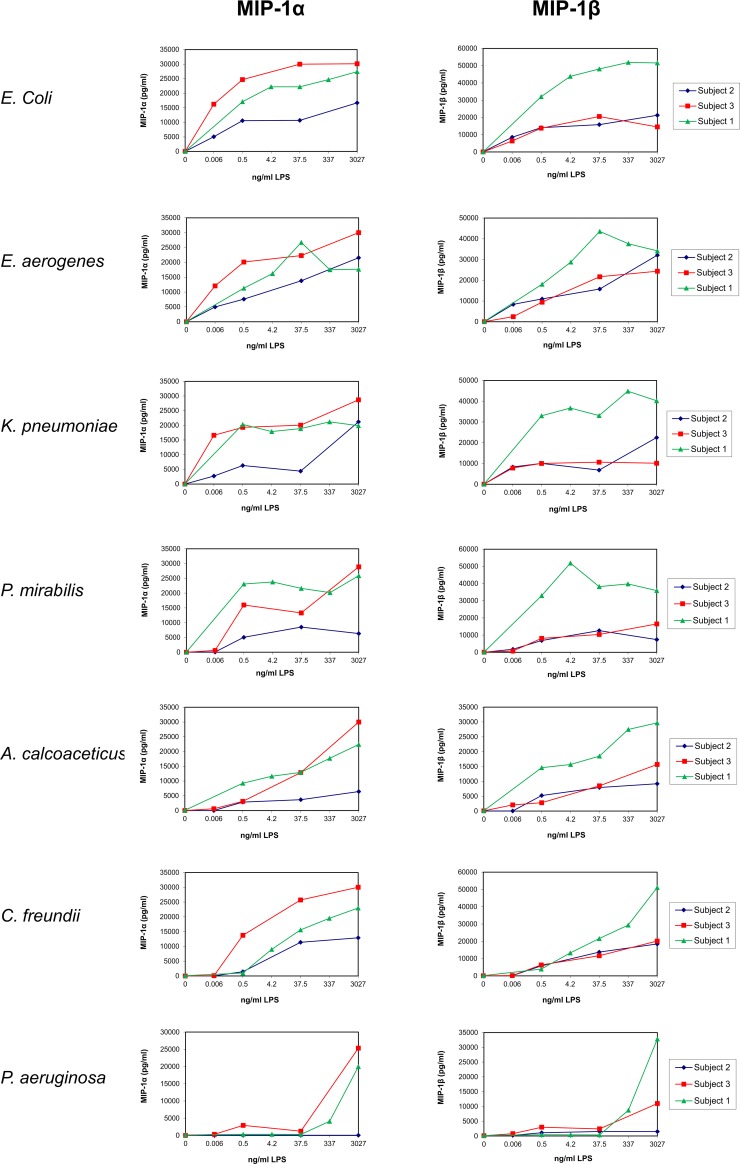
Dose response of LPS on the release of chemokines by UBMC. UBMC isolated from three of the ten deliveries (Subjects 1–3; Table 1) were stimulated for 36 hours with increasing amounts (0–3027 ng/ml) of purified LPS derived from the indicated seven gram-negative bacteria (*E*. *coli*, *E*. *aerogenes*, *K*. *pneumoniae*, *P*. *mirabilis*, *A*. *calcoaceticus*, *C*. *freundii*, *P*. *aeruginosa*). The levels of chemokines (MIP-1α, MIP-1β) released in the supernatants of UBMC were measured by Luminex, as described in the Methods section. Based on the assays presented in Figs 1–4, the concentration of 37.5 ng/ml LPS was used for further experiments.

**Fig 5:**
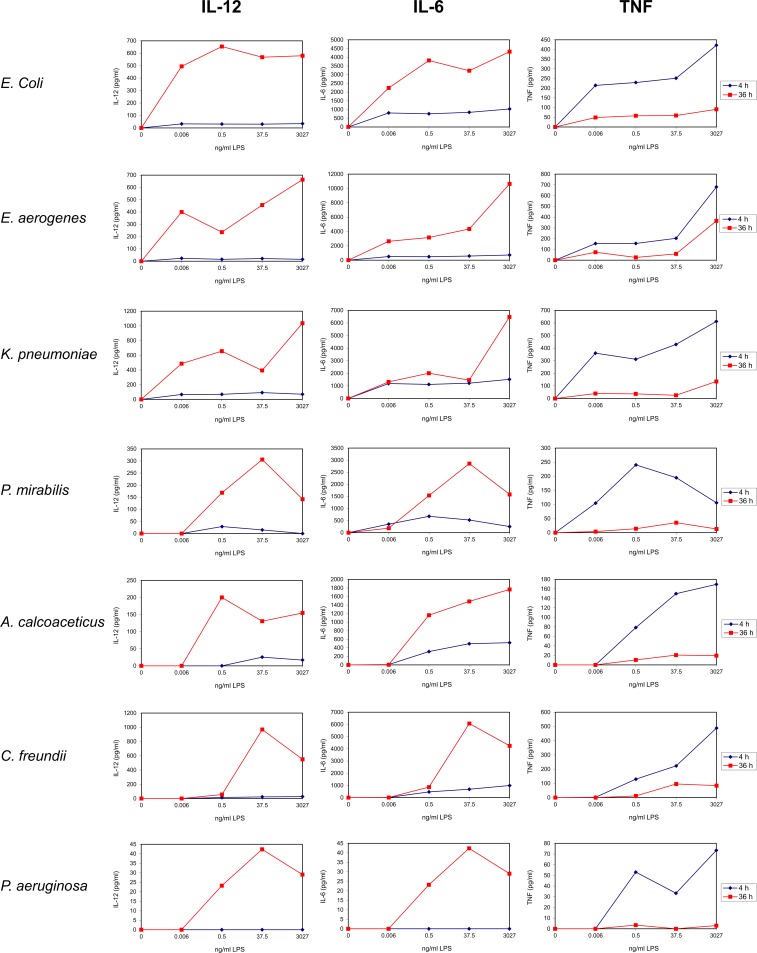
Dose response and time course of LPS on the release of cytokines and chemokines by UBMC. UBMC isolated from Subject 2 (Table 1) were stimulated for either 4 or 36 hours with increasing amounts (0–3027 ng/ml) of purified LPS derived from the indicated seven gram-negative bacteria (*E*. *coli*, *E*. *aerogenes*, *K*. *pneumoniae*, *P*. *mirabilis*, *A*. *calcoaceticus*, *C*. *freundii*, *P*. *aeruginosa*). The levels of IL-12, IL-6 and TNF released in the UBMC supernatant were measured by Luminex, as described in the Methods section. Based on these preliminary assays (and reports from the literature; see text), a stimulation duration of 36 h was used for further experiments.

Pilot experiments were performed on Subjects number 1 to 3 to identify an optimal LPS concentration, duration of stimulation and relevant cytokines (Figs 1–5; Table 2). UBMC of three donors were stimulated for 36 hours with increasing amounts (from 0.006 to 3027 ng/ml) of purified LPS (Figs 1–4). A subanalysis was performed on one donor to compare a 4-hour to a 36-hour LPS stimulation on selected cytokines (Fig 5). Based on these experiments, as well as on reports from the literature [62–65], we selected a LPS concentration of 37.5 ng/ml and a stimulation duration of 36 hours as optimal to detect significant levels of ten selected cytokines and chemokines (IL-1β, IL-10, IL-6, IL-8, IL-12 [p40/p70], TNF, IFN-γ, MIP-1α, MIP-1β, MCP-1). Although the concentration of 37.5 ng/ml LPS was not sufficient to induce a strong cytokine response in some cases (e.g. *P*. *aeruginosa*; Figs 1–4), this working concentration was chosen to remain in a range of LPS close to that normally detected in the plasma of patients with sepsis [66]. Additional secreted cytokines and growth factors measured in the supernatant of one donor using a 30-plex cytokine panel are shown in Table 2. In this donor, low to high levels of secreted factors were detected (low: IL-2, IL-4, IL-5, IL-13, IP-10, eotaxin, HGF; intermediate: IL-15, MIG, FGF-basic; high: IL-7, IL-1RA, IL-2R, RANTES, G-CSF, GM-CSF, VEGF), while IL-17, EGF and IFN-α were undetectable (Table 2).

**Table 2:** Luminex assay using a human cytokine 30-plex panel on UBMC's supernantant of Subject 1, following stimulation for 36 h with 37.5 ng/ml purified LPS obtained from the indicated baterial species (*E*. *coli*, *E*. *aerogenes*, *K*. *pneumoniae*, *P*. *mirabilis*, *A*. *calcoaceticus*, *C*. *freundii*, *P*. *aeruginosa*). Abbreviation: SD, standard deviation.

		*P*. *mirabilis*	*E*. *aerogenes*	*A*. *calcoaceticus*	*C*. *freundii*	*P*. *aeruginosa*	*E*. *coli*	*K*. *pneumoniae*
IL-6	pg/ml	saturated signal	saturated signal	saturated signal	saturated signal	1280	saturated signal	saturated signal
	SD	-	-	-	-	981	-	-
TNF	pg/ml	460	1320	117	105	8.37	2150	1780
	SD	186	205	13	5	5	346	163
IL-1β	pg/ml	1910	2440	945	938	47.2	2730	3060
	SD	354	7	52	315	33	148	28
IL-10	pg/ml	246	345	132	97.1	0	389	318
	SD	78	69	40	37	0	89	93
IL-12 (p40/p70)	pg/ml	3210	3880	1610	1450	71.7	3880	3720
	SD	1004	1457	191	481	39	813	1061
IFN-γ	pg/ml	28.9	26	18.1	19.4	12.9	30.6	31.4
	SD	5	2	0	1	1	6	8
IL-8	pg/ml	26600	24400	19600	25600	23200	22100	18300
	SD	919	3041	5940	2616	1556	212	0
MIP-1α	pg/ml	21600	26700	12900	15600	290	22200	18900
	SD	1909	4525	2616	5586	133	2263	3606
MIP-1β	pg/ml	38200	43600	18500	21600	383	48100	33100
	SD	5798	4596	2616	9687	151	22345	6223
MCP-1	pg/ml	30000	28600	26600	29600	4080	29000	25800
	SD	1414	283	1202	3041	2595	2051	2404
IL-1RA	pg/ml	8050	10500	5350	5690	785	10800	10600
	SD	1245	1464	78	1704	284	990	778
G-CSF	pg/ml	4160	4820	2000	1860	0	5440	4620
	SD	445	877	417	778	0	1110	566
IL-2R	pg/ml	1290	1220	1170	1210	194	1340	1260
	SD	7	57	14	35	0	106	35
VEGF	pg/ml	1100	1040	962	1030	343	1080	1030
	SD	28	7	70	83	170	64	21
RANTES	pg/ml	360	736	142	134	0	744	591
	SD	204	361	37	65	0	336	216
GM-CSF	pg/ml	261	350	133	120	21.1	333	254
	SD	21	26	6	15	8	45	25
IL-7	pg/ml	240	243	225	237	108	239	225
	SD	2	1	17	6	38	8	5
FGF-basic	pg/ml	122	136	114	110	0	115	134
	SD	11	22	2	0	0	0	0
IL-15	pg/ml	81.1	0	0	0	0	77.4	0
	SD	7	0	0	0	0	0	0
MIG	pg/ml	72.1	72.8	46	0	0	58.5	44.1
	SD	4	6	0	0	0	1	3
HGF	pg/ml	35.8	36.3	0	0	0	0	0
	SD	0	0	0	0	0	0	0
IL-13	pg/ml	27.8	34.1	27.8	26.3	0	26.3	26.3
	SD	0	0	0	2	0	2	2
IL-4	pg/ml	18.8	15.9	12.9	14.9	0	16.9	13.9
	SD	0	1	0	6	0	6	4
IP-10	pg/ml	14.9	21	10.4	8.87	6.15	24.8	32.9
	SD	6	3	1	1	2	8	16
IL-5	pg/ml	7.79	11	3.22	3.22	0	10.8	11.3
	SD	2	0	1	1	0	3	3
Eotaxin	pg/ml	4.43	4.49	4.02	4.08	0	4.26	4.14
	SD	0	0	0	0	0	1	0
IL-2	pg/ml	4.97	5.49	0	4.72	0	5.23	4.97
	SD	0	1	0	0	0	0	1
IL-17	pg/ml	0	0	0	0	0	0	0
	SD	0	0	0	0	0	0	0
EGF	pg/ml	below detection	below detection	below detection	below detection	below detection	below detection	below detection
	SD	-	-	-	-	-	-	-
IFN-α	pg/ml	below detection	below detection	below detection	below detection	below detection	below detection	below detection
	SD	-	-	-	-	-	-	-

**Fig 6:**
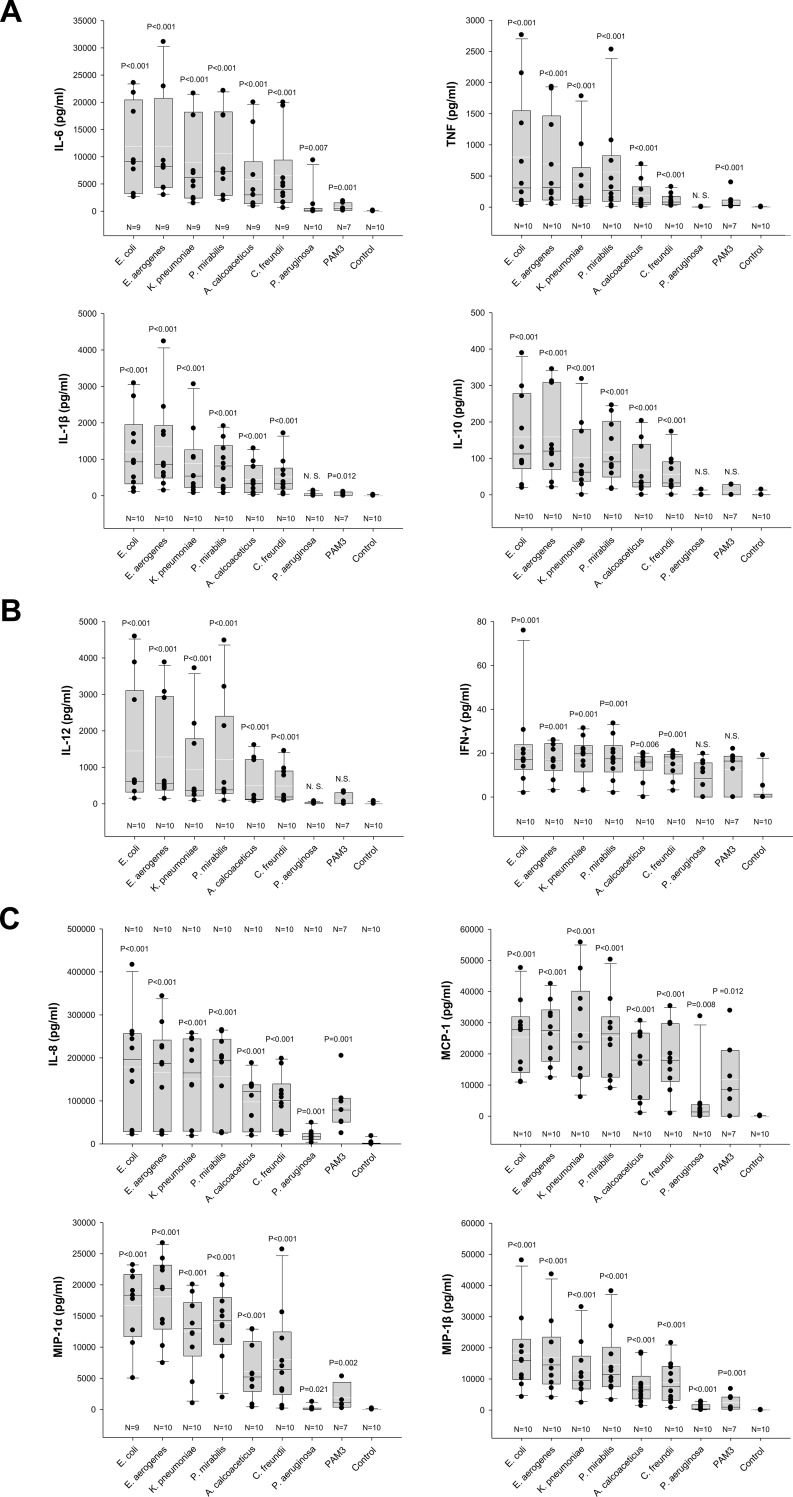
Levels of cytokines secreted by LPS-stimulated UBMC. Levels of pro-inflammatory (IL-6, IL-1ß, TNF) and anti-inflammatory (IL-10) cytokines (**A**), of the TH_1_-polarizing cytokines IL-12 (p40/p70) and IFN-γ (**B**), and of the chemokines IL-8, MIP-1α, MIP-1β and MCP-1 (**C**) released by LPS-stimulated UBMC. UBMC isolated from ten uncomplicated deliveries of at-term newborns were stimulated for 36 h with 37.5 ng/ml purified LPS derived from the indicated seven gram-negative bacteria (*E*. *coli*, *E*. *aerogenes*, *K*. *pneumoniae*, *P*. *mirabilis*, *A*. *calcoaceticus*, *C*. *freundii*, *P*. *aeruginosa*). Unstimulated UBMC served as reference (Control). The TLR1/2 agonist PAM3 served as additional control in seven out of ten donors. Cytokines levels in the respective UBMC supernatants were measured by Luminex. Differences between unstimulated and stimulated conditions were evaluated using the non-parametric Mann-Whitney U-test. Respective P values are indicated above each box plot. Medians (horizontal white line), arithmetic means (horizontal black line) and interquartile ranges are shown. IL-6 levels in Subject 1 were above the detection range after stimulation with six out of seven LPS variants (see also Figs 1–4 and Table 2), and thus the respective concentrations were excluded from the graphic representation and statistical analysis.

Seven additional UBMC preparations were stimulated with 37.5 ng/ml LPS for 36 hours. The same concentration of the synthetic TLR1/TLR2 agonist PAM3 [61] was tested in parallel. Levels of secreted pro-inflammatory cytokines (IL-6, IL-1β, TNF), anti-inflammatory cytokine (IL-10), TH_1_-cytokines (IL-12, IFN-γ), and chemokines (IL-8, MIP-1α, MIP-1β, MCP-1) were measured by Luminex. Results obtained from the ten donors in comparison to the unstimulated control are shown in Fig 6.

Six out of the seven purified LPS (*E*. *coli*, *E*. *aerogenes*, *P*. *mirabilis*, *K*. *pneumoniae*, *A*. *calcoaceticus*, *C*. *freundii*) induced significant levels of the ten cytokines and chemokines investigated (Fig 6A–6C). With the exception of IL-6, LPS from *P*. *aeruginosa* did not induce significant production of pro- and anti-inflammatory cytokines (Fig 6A). It induced however low but significant levels of chemokines by UBMC (Fig 6C). In comparison, the TLR1/2 agonist PAM3 induced no or weak cytokine production (Fig 6A and 6B), and significant chemokine production (Fig 6C).

The donor-to-donor variability in the levels of detected cytokines was high for all tested cytokines and chemokines (Figs 1–4 and 6). For instance, the level of TNF in the UBMC supernatant upon stimulation with LPS from *E*. *coli* ranged from 38 to 2763 pg/ml (median TNF concentration of 309 pg/ml) among the ten donors. Moreover, LPS from different bacterial species induced distinct cytokines profiles in a given UBMC preparation (Figs 1–4 and 6). Among the ten donors, LPS from *E*. *coli* and *E*. *aerogenes* induced the highest median level of cytokines and chemokines, followed by *P*. *mirabilis* and *K*. *pneumoniae*. LPS from *A*. *calcoaceticus* and *C*. *freundii* induced further lower median cytokine and chemokines levels, and–as mentioned above–LPS from *P*. *aeruginosa* was the least active (Fig 6A–6C).

After 36 hours of LPS stimulation, the most prominent detected cytokines were the pro-inflammatory cytokine IL-6 and the chemokines IL-8, MIP-1α, MIP-1β and MCP-1 (median levels >2,900 pg/ml; Fig 6A–6C). TNF, IL-1β, IL-10 and IL-12 were detected at intermediate levels (median between 33 and 929 pg/ml; Fig 6A and 6B), and IFN-γ was the lowest (median level ≤20 pg/ml in response to all LPS variants; Fig 6B).

## Discussion

We describe here the profile of cytokines and chemokines released by UBMC following their *in vitro* stimulation by LPS of seven common vaginal gram-negative bacteria species. Four groups of secreted factors were investigated: pro-inflammatory cytokines (IL-6, IL-1β, TNF), an anti-inflammatory cytokine (IL-10), TH_1_-type cytokines (IL-12, IFN-γ) and chemokines (IL-8, MIP-1α, MIP-1β, MCP-1). Herein, we could confirm our hypothesis that LPS of different gram-negative bacterial species induce distinct inflammatory responses and thus might exert different immunomodulatory functions. We showed that *in vitro* stimulation of freshly isolated UBMC with purified LPS of these bacteria induced different levels of secreted pro- and anti-inflammatory cytokines and chemokines. Interestingly, the range of cytokine and chemokine concentrations detected in the supernatants of stimulated UBMC varied drastically between donors, indicating major differences in individual responsiveness to a given LPS variant.

**Fig 7:**
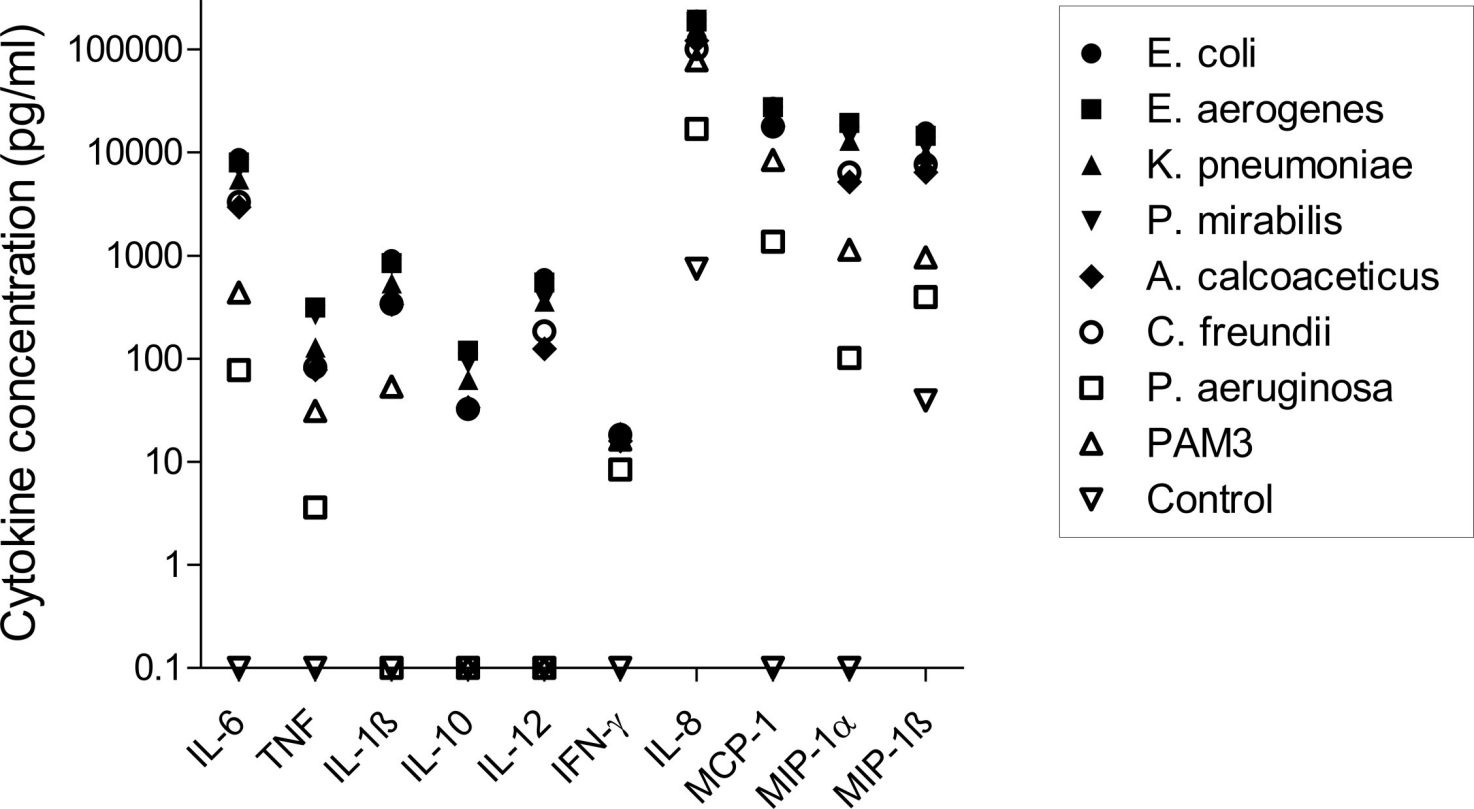
Summary of the cytokine profiles in UBMC stimulated with LPS of seven gram-negative bacteria. Median cytokine levels in LPS-stimulated UBMC isolated from ten uncomplicated deliveries. Median cytokine levels are directly derived from the data presented in Fig 6. Due to log scale representation, values of 0 pg/ml were replaced by 0.1; thus values depicted at 0.1 pg/ml along the x axis are actually equal to zero. IL-8, IL-6, MIP-1α, MIP-1β and MCP-1 showed the highest median level of induction, followed by TNF, IL-1β, IL-10 and IL-12. IFN-γ showed the lowest median level of secretion.

All ten cytokines and chemokines were induced in UBMC after 36 hours stimulation with the different LPS, albeit at different levels, as summarized in Fig 7.

The pro-inflammatory cytokine IL-6 and the chemokines IL-8, MIP-1α, MIP-1β and MCP-1 showed the highest induction, with median levels ranging from 77 to 196,000 pg/ml upon LPS stimulation. Elevated IL-6 and IL-8 levels after LPS stimulation of umbilical cord blood was also reported by others [49,51,67]. Our results thus support the observation that intrauterine infection results in the secretion of pro-inflammatory cytokines and chemokines such as Il-1ß, Il-6, IL-8, TNF and MCP-1, in association with PTL and fetal brain injury [23,36,39,68–70].

The anti-inflammatory and TH_2_-polarizing cytokine IL-10 was induced at low levels (median 0 to 120 pg/ml) by all seven LPS tested. IL-10 is an important regulator and suppressor of the pro-inflammatory cascade [36]. IL-10 is normally produced at the maternal-fetal interface, and exerts together with progesterone a strong immunomodulatory protection of the fetal allograft [36].

IL-12 is an essential cytokine for the generation of the TH_1_-type immune responses. It is produced by monocytes and dendritic cells (DC) upon stimulation with LPS [71]. In our study median IL-12 (p40/p70) levels ranged from 126 to 605 pg/ml in the supernatants of LPS-stimulated UBMC, with maximum levels ranging from 1,450 and 4,593 pg/ml between LPS variants. Others showed that IL-12 is weakly expressed and secreted in LPS-stimulated whole cord blood [49,72].

As opposed to IL-12, levels of the TH_1_ cytokine IFN-γ were consistently low in all donors and upon stimulation with all seven LPS. This is in line with published report in LPS-stimulated PBMC [73]. However, we cannot exclude that a different duration of LPS stimulation might allow a better detection of secreted IFN-γ levels. For instance, the level of secreted TNF in Subject 2 was higher after 4 hours of LPS stimulation with all seven LPS variants than after 36 hours.

Other studies compared the response to LPS of UBMC to that of adult peripheral blood mononuclear cells. Some studies showed a reduced cytokine production (e.g. IL-12) in LPS-stimulated whole cord blood compared to adult whole blood [49,72], supporting the proposition that TLR4-mediated response is impaired in neonates and infants [50,52,72]. Other studies however described an elevated cytokine responses, in particular of IL-6, TNF, IL-8 and IL-10 [49,51,67], and even a newborn-specific cytokine production [74] after LPS stimulation of umbilical cord blood. These differences in reported LPS responsiveness might be a consequence of differential TLR4 regulation, due to differences in TLR4 expression in neonates and adults [49,51,75] and/or due to TLR4 gene polymorphism [76].

Alternatively, these functional differences in LPS responsiveness might also be due to the use of LPS of different origin and species in these reported studies. Indeed, in our study the comparison of the stimulatory activity of purified LPS from seven common gram-negative bacteria species revealed functional differences. LPS of *E*. *coli* and *E*. *aerogenes* provoked the highest cytokine and chemokine release, while LPS of *P*. *aeruginosa* was the least active. The low stimulatory activity of LPS of *P*. *aeruginosa* might be explained by its reduced acylation state, which is an important component of TLR4 recognition, compared to other LPS [47]. The observed differences in LPS activity therefore confirm our hypothesis that different LPS molecules potentially mediate different inflammatory responses, in line with reports from the literature [44,46–48]. Our data are also in agreement with the virulence and neonatal complications associated with *E*. *coli*, *including neonatal sepsis* [77–79] and with *E*. *aerogenes* infections [80,81].

In addition, we observed striking differences in the responsiveness of the ten UBMC preparations, resulting in skewed levels of cytokine release. Higher cytokine release in some donors did not correlate with longer cord blood storage before UBMC isolation (Fig 5 and Table 1). Therefore higher cytokine release cannot be accounted for artefactual cytokine release by contaminating granulocytes, which are known to accumulate in the mononuclear cell layer during density gradient centrifugation in case of delayed blood processing [82,83]. The reason for such variability in cytokine levels is unknown and remains to be investigated. It might be the consequence of differences in TLR4 function between donors, due to differential gene expression and/or polymorphism [75,76]. Nevertheless, the observation that UBMC of neonates born after uncomplicated gestation and delivery produce such variable levels of cytokines upon LPS stimulation raises the possibility that pregnant women might experience different levels of inflammatory response following a bacterial infection, potentially differentially affecting pregnancy outcome. It would be interesting to compare these results to those generated from UBMC of premature neonates with a declared bacterial infection.

Prevention of infection remains the most promising approach to reduce PTL and associated mortality and morbidity in this highly vulnerable neonatal population. Diagnostic and treatment of prenatal and neonatal infections is a major challenge. The present study suggests that measuring cytokine levels might guide the diagnosis of bacterial infections. Interestingly, others showed that it is possible to measure cytokine levels in amniotic fluid collected noninvasively from vaginal secretions of women with preterm premature rupture of membranes. Increased levels of amniotic fluid IL-6 and TNF were good predictors of fetal inflammatory response syndrome [84]. By using the umbilical cord as a noninvasive source of neonatal blood, as in our study, one may be able to establish the cytokine profile of neonates and evaluate their risk of infection-related complications after birth.

In terms of treatment of prenatal and neonatal infections, antibiotics acting against gram-negative bacteria exist. However, antenatal antibiotic treatment alone has limited success at preventing PTL or improving neonatal outcome [85]. A combination of anti-inflammatory therapy and effective antibiotics has been proposed to combat intra-uterine infection and reduce associated inflammatory responses leading to PTL and adverse fetal sequelae. Optimal treatments also increase the survival of premature infants by promoting lung maturation, as illustrated by the combination of betamethasone with tocolytic agents such as nonsteroidal anti-inflammatory drugs (NSAIDs) like indomethacin. The anti-inflammatory effect of the combination therapy of betamethasone with indomethacin might also reduce bacterial clearance. Our group showed furthermore that, similar to betamethasone alone, the combination therapy effectively reduced the expression of the pro-inflammatory cytokines TNF, IL-6 and IL-12 [86]. Other studies even suggested that a postnatal application of indomethacin to preterm neonates delivered before 28 weeks of gestation provided beneficial effects on the fetal brain [87]. This neuroprotective effects of indomethacin might be due to an increase of cerebral vascular resistance and stability of cerebral hemodynamics [88,89]. Additionally to NSAIDs, cytokine suppressive anti-inflammatory drugs (CSAIDs) are novel compounds that specifically target cytokine signaling pathways, exerting anti-inflammatory actions in both human fetal membranes *in vitro* and animal models of intrauterine infection. These compounds have the potential to be safer and more effective than less selective inhibitors of inflammation [90].

In conclusion, we showed that LPS of different vaginal gram-negative bacteria trigger different cytokine and chemokine responses and that individual UBMC differentially respond to LPS stimulation. Further studies are needed to better understand the relationship between intrauterine infection, inflammatory response and PTL, and to develop clinical approaches to reduce preterm delivery and associated neonatal complications.

## References

[1] Goldenberg RL. The management of preterm labor. Obstet Gynecol. 2002;100: 1020–1037. 10.1016/s0029-7844(02)02212-3 12423870

[2] Manktelow BN, Draper ES, Annamalai S, Field D. Factors affecting the incidence of chronic lung disease of prematurity in 1987, 1992, and 1997. Arch Dis Child Fetal Neonatal Ed. 2001;85: F33–35. 10.1136/fn.85.1.F33 11420319 PMC1721286

[3] Menon R, Fortunato SJ. Fetal membrane inflammatory cytokines: a switching mechanism between the preterm premature rupture of the membranes and preterm labor pathways. J Perinat Med. 2004;32: 391–399. 10.1515/JPM.2004.134 15493713

[4] Walker KF, Thornton JG. Tocolysis and preterm labour. Lancet. 2016;387: 2068–2070. 10.1016/S0140-6736(16)00590-0 26944025

[5] Wood NS, Marlow N, Costeloe K, Gibson AT, Wilkinson AR. Neurologic and developmental disability after extremely preterm birth. EPICure Study Group. N Engl J Med. 2000;343: 378–384. 10.1056/NEJM200008103430601 10933736

[6] Martin JA, Hamilton BE, Osterman MJK, Driscoll AK, Drake P. Births: Final Data for 2016. Natl Vital Stat Rep. 2018;67: 1–55. 29775434

[7] Chawanpaiboon S, Vogel JP, Moller A-B, Lumbiganon P, Petzold M, Hogan D, et al Global, regional, and national estimates of levels of preterm birth in 2014: a systematic review and modelling analysis. The Lancet Global Health. 2019;7: e37–e46. 10.1016/S2214-109X(18)30451-0 30389451 PMC6293055

[8] Lettieri L, Vintzileos AM, Rodis JF, Albini SM, Salafia CM. Does “idiopathic” preterm labor resulting in preterm birth exist? Am J Obstet Gynecol. 1993;168: 1480–1485. 10.1016/s0002-9378(11)90785-6 8498431

[9] Liu L, Johnson HL, Cousens S, Perin J, Scott S, Lawn JE, et al Global, regional, and national causes of child mortality: an updated systematic analysis for 2010 with time trends since 2000. Lancet. 2012;379: 2151–2161. 10.1016/S0140-6736(12)60560-1 22579125

[10] Romero R, Gómez R, Chaiworapongsa T, Conoscenti G, Kim JC, Kim YM. The role of infection in preterm labour and delivery. Paediatr Perinat Epidemiol. 2001;15 Suppl 2: 41–56. 11520399 10.1046/j.1365-3016.2001.00007.x

[11] Hillier SL, Nugent RP, Eschenbach DA, Krohn MA, Gibbs RS, Martin DH, et al Association between bacterial vaginosis and preterm delivery of a low-birth-weight infant. The Vaginal Infections and Prematurity Study Group. N Engl J Med. 1995;333: 1737–1742. 10.1056/NEJM199512283332604 7491137

[12] Pankuch GA, Appelbaum PC, Lorenz RP, Botti JJ, Schachter J, Naeye RL. Placental microbiology and histology and the pathogenesis of chorioamnionitis. Obstet Gynecol. 1984;64: 802–806. 6390279

[13] Hillier SL, Martius J, Krohn M, Kiviat N, Holmes KK, Eschenbach DA. A case-control study of chorioamnionic infection and histologic chorioamnionitis in prematurity. N Engl J Med. 1988;319: 972–978. 10.1056/NEJM198810133191503 3262199

[14] Soraisham AS, Singhal N, McMillan DD, Sauve RS, Lee SK, Canadian Neonatal Network. A multicenter study on the clinical outcome of chorioamnionitis in preterm infants. Am J Obstet Gynecol. 2009;200: 372.e1–6. 10.1016/j.ajog.2008.11.034 19217596

[15] Martinelli P, Sarno L, Maruotti GM, Paludetto R. Chorioamnionitis and prematurity: a critical review. J Matern Fetal Neonatal Med. 2012;25 Suppl 4: 29–31. 10.3109/14767058.2012.714981 22958008

[16] García-Muñoz Rodrigo F, Galán Henríquez GM, Ospina CG. Morbidity and mortality among very-low-birth-weight infants born to mothers with clinical chorioamnionitis. Pediatr Neonatol. 2014;55: 381–386. 10.1016/j.pedneo.2013.12.007 24745649

[17] Romero R, Yoon BH, Chaemsaithong P, Cortez J, Park C-W, Gonzalez R, et al Secreted phospholipase A2 is increased in meconium-stained amniotic fluid of term gestations: potential implications for the genesis of meconium aspiration syndrome. J Matern Fetal Neonatal Med. 2014;27: 975–983. 10.3109/14767058.2013.847918 24063538 PMC5891099

[18] Cooke R. Chorioamnionitis, maternal fever, and neonatal encephalopathy. Dev Med Child Neurol. 2008;50: 9 10.1111/j.1469-8749.2007.00009.x 18173621

[19] Korzeniewski SJ, Romero R, Cortez J, Pappas A, Schwartz AG, Kim CJ, et al A “multi-hit” model of neonatal white matter injury: cumulative contributions of chronic placental inflammation, acute fetal inflammation and postnatal inflammatory events. J Perinat Med. 2014;42: 731–743. 10.1515/jpm-2014-0250 25205706 PMC5987202

[20] Pappas A, Kendrick DE, Shankaran S, Stoll BJ, Bell EF, Laptook AR, et al Chorioamnionitis and early childhood outcomes among extremely low-gestational-age neonates. JAMA Pediatr. 2014;168: 137–147. 10.1001/jamapediatrics.2013.4248 24378638 PMC4219500

[21] Shatrov JG, Birch SCM, Lam LT, Quinlivan JA, McIntyre S, Mendz GL. Chorioamnionitis and cerebral palsy: a meta-analysis. Obstet Gynecol. 2010;116: 387–392. 10.1097/AOG.0b013e3181e90046 20664400

[22] Romero R, Dey SK, Fisher SJ. Preterm labor: one syndrome, many causes. Science. 2014;345: 760–765. 10.1126/science.1251816 25124429 PMC4191866

[23] Dammann O, Leviton A. Maternal intrauterine infection, cytokines, and brain damage in the preterm newborn. Pediatr Res. 1997;42: 1–8. 10.1203/00006450-199707000-00001 9212029

[24] Leviton A. Preterm birth and cerebral palsy: is tumor necrosis factor the missing link? Dev Med Child Neurol. 1993;35: 553–558. 10.1111/j.1469-8749.1993.tb11688.x 8504899

[25] Abdallah MW, Larsen N, Mortensen EL, Atladóttir HÓ, Nørgaard-Pedersen B, Bonefeld-Jørgensen EC, et al Neonatal levels of cytokines and risk of autism spectrum disorders: an exploratory register-based historic birth cohort study utilizing the Danish Newborn Screening Biobank. J Neuroimmunol. 2012;252: 75–82. 10.1016/j.jneuroim.2012.07.013 22917523

[26] Sørensen HJ, Mortensen EL, Reinisch JM, Mednick SA. Association between prenatal exposure to bacterial infection and risk of schizophrenia. Schizophr Bull. 2009;35: 631–637. 10.1093/schbul/sbn121 18832344 PMC2669577

[27] Kassim Z, Aziz AA, Haque QM, Cheung HAS. Isolation of Proteus mirabilis from severe neonatal sepsis and central nervous system infection with extensive pneumocephalus. Eur J Pediatr. 2003;162: 644–645. 10.1007/s00431-003-1240-9 12836017

[28] Viswanathan R, Singh AK, Mukherjee S, Mukherjee R, Das P, Basu S. An outbreak of neonatal sepsis presenting with exanthematous rash caused by Klebsiella pneumoniae. Epidemiol Infect. 2011;139: 226–228. 10.1017/S0950268810000701 20370956

[29] Rodrigues J, Rocha D, Santos F, João A. Neonatal Citrobacter koseri Meningitis: Report of Four Cases. Case Rep Pediatr. 2014;2014: 195204 10.1155/2014/195204 24716069 PMC3971854

[30] Shete VB, Ghadage DP, Muley VA, Bhore AV. Acinetobacter septicemia in neonates admitted to intensive care units. J Lab Physicians. 2009;1: 73–76. 10.4103/0974-2727.59704 21938255 PMC3167973

[31] Ovalle A, Martínez MA, Kakarieka E, García M, Salinas A. [Fatal neonatal sepsis caused by vertical transmission of Morganella morganii. Report of one case]. Rev Med Chil. 2009;137: 1201–1204. doi: /S0034-98872009000900010 20011962

[32] Schulz D, Schlieckau F, Fill Malfertheiner S, Reuschel E, Seelbach-Göbel B, Ernst W. Effect of betamethasone, indomethacin and fenoterol on neonatal and maternal mononuclear cells stimulated with Escherichia coli. Cytokine. 2019;116: 97–105. 10.1016/j.cyto.2018.12.017 30703694

[33] Rietschel ET, Kirikae T, Schade FU, Mamat U, Schmidt G, Loppnow H, et al Bacterial endotoxin: molecular relationships of structure to activity and function. FASEB J. 1994;8: 217–225. 10.1096/fasebj.8.2.8119492 8119492

[34] Raetz CRH, Whitfield C. Lipopolysaccharide endotoxins. Annu Rev Biochem. 2002;71: 635–700. 10.1146/annurev.biochem.71.110601.135414 12045108 PMC2569852

[35] Lu Y-C, Yeh W-C, Ohashi PS. LPS/TLR4 signal transduction pathway. Cytokine. 2008;42: 145–151. 10.1016/j.cyto.2008.01.006 18304834

[36] Peltier MR. Immunology of term and preterm labor. Reprod Biol Endocrinol. 2003;1: 122 10.1186/1477-7827-1-122 14651749 PMC305338

[37] O’Hern Perfetto C, Fan X, Dahl S, Krieg S, Westphal LM, Bunker Lathi R, et al Expression of interleukin-22 in decidua of patients with early pregnancy and unexplained recurrent pregnancy loss. J Assist Reprod Genet. 2015;32: 977–984. 10.1007/s10815-015-0481-7 25925347 PMC4491088

[38] Al-Amin MM, Alam T, Hasan SMN, Hasan AT, Quddus AHMR. Prenatal maternal lipopolysaccharide administration leads to age- and region-specific oxidative stress in the early developmental stage in offspring. Neuroscience. 2016;318: 84–93. 10.1016/j.neuroscience.2016.01.002 26774051

[39] Menon R, Peltier MR, Eckardt J, Fortunato SJ. Diversity in cytokine response to bacteria associated with preterm birth by fetal membranes. Am J Obstet Gynecol. 2009;201: 306.e1–6. 10.1016/j.ajog.2009.06.027 19733282

[40] Berthet J, Damien P, Hamzeh-Cognasse H, Arthaud C-A, Eyraud M-A, Zéni F, et al Human platelets can discriminate between various bacterial LPS isoforms via TLR4 signaling and differential cytokine secretion. Clin Immunol. 2012;145: 189–200. 10.1016/j.clim.2012.09.004 23108090

[41] Schildberger A, Rossmanith E, Eichhorn T, Strassl K, Weber V. Monocytes, peripheral blood mononuclear cells, and THP-1 cells exhibit different cytokine expression patterns following stimulation with lipopolysaccharide. Mediators Inflamm. 2013;2013: 697972 10.1155/2013/697972 23818743 PMC3681313

[42] Di Simone N, Di Nicuolo F, Marana R, Castellani R, Ria F, Veglia M, et al Synthetic PreImplantation Factor (PIF) prevents fetal loss by modulating LPS induced inflammatory response. PLoS ONE. 2017;12: e0180642 10.1371/journal.pone.0180642 28704412 PMC5507516

[43] Mizoguchi M, Ishida Y, Nosaka M, Kimura A, Kuninaka Y, Yahata T, et al Prevention of lipopolysaccharide-induced preterm labor by the lack of CX3CL1-CX3CR1 interaction in mice. PLoS ONE. 2018;13: e0207085 10.1371/journal.pone.0207085 30399192 PMC6219809

[44] Gangloff SC, Hijiya N, Haziot A, Goyert SM. Lipopolysaccharide structure influences the macrophage response via CD14-independent and CD14-dependent pathways. Clin Infect Dis. 1999;28: 491–496. 10.1086/515176 10194066

[45] Netea MG, van Deuren M, Kullberg BJ, Cavaillon J-M, Van der Meer JWM. Does the shape of lipid A determine the interaction of LPS with Toll-like receptors? Trends Immunol. 2002;23: 135–139. 11864841 10.1016/s1471-4906(01)02169-x

[46] Beutler B., Inferences questions and possibilities in Toll-like receptor signalling. Nature. 2004;430: 257–263. 10.1038/nature02761 15241424

[47] Hajjar AM, Ernst RK, Tsai JH, Wilson CB, Miller SI. Human Toll-like receptor 4 recognizes host-specific LPS modifications. Nat Immunol. 2002;3: 354–359. 10.1038/ni777 11912497

[48] Maeshima N, Fernandez RC. Recognition of lipid A variants by the TLR4-MD-2 receptor complex. Front Cell Infect Microbiol. 2013;3: 3 10.3389/fcimb.2013.00003 23408095 PMC3569842

[49] Yerkovich ST, Wikström ME, Suriyaarachchi D, Prescott SL, Upham JW, Holt PG. Postnatal development of monocyte cytokine responses to bacterial lipopolysaccharide. Pediatr Res. 2007;62: 547–552. 10.1203/PDR.0b013e3181568105 17805207

[50] Levy O, Coughlin M, Cronstein BN, Roy RM, Desai A, Wessels MR. The adenosine system selectively inhibits TLR-mediated TNF-alpha production in the human newborn. J Immunol. 2006;177: 1956–1966. 10.4049/jimmunol.177.3.1956 16849509 PMC2881468

[51] Sugitharini V, Pavani K, Prema A, Berla Thangam E. TLR-mediated inflammatory response to neonatal pathogens and co-infection in neonatal immune cells. Cytokine. 2014;69: 211–217. 10.1016/j.cyto.2014.06.003 24999538

[52] Sadeghi K, Berger A, Langgartner M, Prusa A-R, Hayde M, Herkner K, et al Immaturity of infection control in preterm and term newborns is associated with impaired toll-like receptor signaling. J Infect Dis. 2007;195: 296–302. 10.1086/509892 17191175

[53] Seelbach-Goebel B. Antibiotic Therapy for Premature Rupture of Membranes and Preterm Labor and Effect on Fetal Outcome. Geburtshilfe Frauenheilkd. 2013;73: 1218–1227. 10.1055/s-0033-1360195 24771902 PMC3964356

[54] Haraguchi GE, Zähringer U, Jann B, Jann K, Hull RA, Hull SI. Genetic characterization of the O4 polysaccharide gene cluster from Escherichia coli. Microb Pathog. 1991;10: 351–361. 1721674 10.1016/0882-4010(91)90080-t

[55] Bryn K, Rietschel ET. L-2-hydroxytetradecanoic acid as a constituent of Salmonella lipopolysaccharides (lipid A). Eur J Biochem. 1978;86: 311–315. 10.1111/j.1432-1033.1978.tb12312.x 658049

[56] Sidorczyk Z, Zähringer U, Rietschel ET. Chemical structure of the lipid A component of the lipopolysaccharide from a Proteus mirabilis Re-mutant. Eur J Biochem. 1983;137: 15–22. 10.1111/j.1432-1033.1983.tb07789.x 6360683

[57] Süsskind M, Müller-Loennies S, Nimmich W, Brade H, Holst O. Structural investigation on the carbohydrate backbone of the lipopolysaccharide from Klebsiella pneumoniae rough mutant R20/O1-. Carbohydr Res. 1995;269: C1–7. 10.1016/0008-6215(95)00002-b 7773983

[58] Kawahara K, Brade H, Rietschel ET, Zähringer U. Studies on the chemical structure of the core-lipid A region of the lipopolysaccharide of Acinetobacter calcoaceticus NCTC 10305. Detection of a new 2-octulosonic acid interlinking the core oligosaccharide and lipid A component. Eur J Biochem. 1987;163: 489–495. 10.1111/j.1432-1033.1987.tb10895.x 3830168

[59] Brandenburg K, Heinbockel L, Correa W, Fukuoka S, Gutsmann T, Zähringer U, et al Supramolecular structure of enterobacterial wild-type lipopolysaccharides (LPS), fractions thereof, and their neutralization by Pep19-2.5. J Struct Biol. 2016;194: 68–77. 10.1016/j.jsb.2016.01.014 26828112

[60] Kooistra O, Bedoux G, Brecker L, Lindner B, Sánchez Carballo P, Haras D, et al Structure of a highly phosphorylated lipopolysaccharide core in the Delta algC mutants derived from Pseudomonas aeruginosa wild-type strains PAO1 (serogroup O5) and PAC1R (serogroup O3). Carbohydr Res. 2003;338: 2667–2677. 10.1016/j.carres.2003.07.004 14670725

[61] Ozinsky A, Underhill DM, Fontenot JD, Hajjar AM, Smith KD, Wilson CB, et al The repertoire for pattern recognition of pathogens by the innate immune system is defined by cooperation between toll-like receptors. Proc Natl Acad Sci USA. 2000;97: 13766–13771. 10.1073/pnas.250476497 11095740 PMC17650

[62] Eggesbø JB, Hjermann I, Lund PK, Joø GB, Ovstebø R, Kierulf P. LPS-induced release of IL-1 beta, IL-6, IL-8, TNF-alpha and sCD14 in whole blood and PBMC from persons with high or low levels of HDL-lipoprotein. Cytokine. 1994;6: 521–529. 7530060 10.1016/1043-4666(94)90080-9

[63] Eggesbø JB, Hjermann I, Joø GB, Ovstebø R, Kierulf P. LPS-induced release of EGF, GM-CSF, GRO alpha, LIF, MIP-1 alpha and PDGF-AB in PBMC from persons with high or low levels of HDL lipoprotein. Cytokine. 1995;7: 562–567. 10.1006/cyto.1995.0076 8580373

[64] Lindner H, Holler E, Ertl B, Multhoff G, Schreglmann M, Klauke I, et al Peripheral blood mononuclear cells induce programmed cell death in human endothelial cells and may prevent repair: role of cytokines. Blood. 1997;89: 1931–1938. 9058713

[65] Kumolosasi E, Salim E, Jantan I, Ahmad W. Kinetics of Intracellular, Extracellular and Production of Pro-Inflammatory Cytokines in Lipopolysaccharide- Stimulated Human Peripheral Blood Mononuclear Cells. Tropical Journal of Pharmaceutical Research. 2014;13: 536–543–543. 10.4314/tjpr.v13i4.8

[66] del Campo R, Martínez E, del Fresno C, Alenda R, Gómez-Piña V, Fernández-Ruíz I, et al Translocated LPS might cause endotoxin tolerance in circulating monocytes of cystic fibrosis patients. PLoS ONE. 2011;6: e29577 10.1371/journal.pone.0029577 22216320 PMC3247277

[67] Dembinski J, Behrendt D, Reinsberg J, Bartmann P. Endotoxin-stimulated production of IL-6 and IL-8 is increased in short-term cultures of whole blood from healthy term neonates. Cytokine. 2002;18: 116–119. 12096927 10.1006/cyto.2002.0880

[68] Hagberg H, Mallard C, Jacobsson B. Role of cytokines in preterm labour and brain injury. BJOG. 2005;112 Suppl 1: 16–18. 10.1111/j.1471-0528.2005.00578.x 15715588

[69] Elovitz MA, Brown AG, Breen K, Anton L, Maubert M, Burd I. Intrauterine inflammation, insufficient to induce parturition, still evokes fetal and neonatal brain injury. Int J Dev Neurosci. 2011;29: 663–671. 10.1016/j.ijdevneu.2011.02.011 21382466 PMC3140629

[70] Gotsch F, Romero R, Kusanovic JP, Mazaki-Tovi S, Pineles BL, Erez O, et al The fetal inflammatory response syndrome. Clin Obstet Gynecol. 2007;50: 652–683. 10.1097/GRF.0b013e31811ebef6 17762416

[71] Usluoglu N, Pavlovic J, Moelling K, Radziwill G. RIP2 mediates LPS-induced p38 and IkappaBalpha signaling including IL-12 p40 expression in human monocyte-derived dendritic cells. Eur J Immunol. 2007;37: 2317–2325. 10.1002/eji.200636388 17578844

[72] Belderbos ME, van Bleek GM, Levy O, Blanken MO, Houben ML, Schuijff L, et al Skewed pattern of Toll-like receptor 4-mediated cytokine production in human neonatal blood: low LPS-induced IL-12p70 and high IL-10 persist throughout the first month of life. Clin Immunol. 2009;133: 228–237. 10.1016/j.clim.2009.07.003 19648060 PMC2892115

[73] Janský L, Reymanová P, Kopecký J. Dynamics of cytokine production in human peripheral blood mononuclear cells stimulated by LPS or infected by Borrelia. Physiol Res. 2003;52: 593–598. 14535835

[74] Mathias B, Mira JC, Rehfuss JP, Rincon JC, Ungaro R, Nacionales DC, et al LPS Stimulation of Cord Blood Reveals a Newborn-Specific Neutrophil Transcriptomic Response and Cytokine Production. Shock. 2017;47: 606–614. 10.1097/SHK.0000000000000800 28410545 PMC5407294

[75] Jaekal J, Abraham E, Azam T, Netea MG, Dinarello CA, Lim J-S, et al Individual LPS responsiveness depends on the variation of toll-like receptor (TLR) expression level. J Microbiol Biotechnol. 2007;17: 1862–1867. 18092472

[76] Michel O, LeVan TD, Stern D, Dentener M, Thorn J, Gnat D, et al Systemic responsiveness to lipopolysaccharide and polymorphisms in the toll-like receptor 4 gene in human beings. J Allergy Clin Immunol. 2003;112: 923–929. 10.1016/j.jaci.2003.05.001 14610481

[77] Krohn MA, Thwin SS, Rabe LK, Brown Z, Hillier SL. Vaginal colonization by Escherichia coli as a risk factor for very low birth weight delivery and other perinatal complications. J Infect Dis. 1997;175: 606–610. 10.1093/infdis/175.3.606 9041332

[78] Watt S, Lanotte P, Mereghetti L, Moulin-Schouleur M, Picard B, Quentin R. Escherichia coli strains from pregnant women and neonates: intraspecies genetic distribution and prevalence of virulence factors. J Clin Microbiol. 2003;41: 1929–1935. 10.1128/JCM.41.5.1929-1935.2003 12734229 PMC154741

[79] Stoll BJ, Hansen NI, Sánchez PJ, Faix RG, Poindexter BB, Van Meurs KP, et al Early onset neonatal sepsis: the burden of group B Streptococcal and E. coli disease continues. Pediatrics. 2011;127: 817–826. 10.1542/peds.2010-2217 21518717 PMC3081183

[80] Burnichon G, Le Floch MF, Virmaux M, Baron R, Tandé D, Lejeune B. [Outbreak of Enterobacter aerogenes in paediatric unit]. Med Mal Infect. 2004;34: 166–170. 15619887 10.1016/j.medmal.2003.12.007

[81] Rosmanova R, Kanovska E, Sredkova M, Khitsova S, Vlkova A. Nosocomial enterobacter—sepsis in neonatal intensive care unit in Pleven. Akush Ginekol (Sofia). 2000;39: 19–22. 11187988

[82] McKenna KC, Beatty KM, Vicetti Miguel R, Bilonick RA. Delayed processing of blood increases the frequency of activated CD11b+ CD15+ granulocytes which inhibit T cell function. J Immunol Methods. 2009;341: 68–75. 10.1016/j.jim.2008.10.019 19041316

[83] Naegelen I, Beaume N, Plançon S, Schenten V, Tschirhart EJ, Bréchard S. Regulation of Neutrophil Degranulation and Cytokine Secretion: A Novel Model Approach Based on Linear Fitting. J Immunol Res. 2015;2015: 817038 10.1155/2015/817038 26579547 PMC4633572

[84] Kunze M, Klar M, Morfeld CA, Thorns B, Schild RL, Markfeld-Erol F, et al Cytokines in noninvasively obtained amniotic fluid as predictors of fetal inflammatory response syndrome. Am J Obstet Gynecol. 2016;215: 96e1–8. 10.1016/j.ajog.2016.01.181 26829512

[85] Brocklehurst P, Gordon A, Heatley E, Milan SJ. Antibiotics for treating bacterial vaginosis in pregnancy. Cochrane Database Syst Rev. 2013; CD000262 10.1002/14651858.CD000262.pub4 23440777 PMC11307253

[86] Ernst W, Kusi E, Fill Malfertheiner S, Reuschel E, Deml L, Seelbach-Göbel B. The effect of Indomethacin and Betamethasone on the cytokine response of human neonatal mononuclear cells to gram-positive bacteria. Cytokine. 2015;73: 91–100. 10.1016/j.cyto.2015.01.023 25743243

[87] Miller SP, Mayer EE, Clyman RI, Glidden DV, Hamrick SEG, Barkovich AJ. Prolonged indomethacin exposure is associated with decreased white matter injury detected with magnetic resonance imaging in premature newborns at 24 to 28 weeks’ gestation at birth. Pediatrics. 2006;117: 1626–1631. 10.1542/peds.2005-1767 16651316

[88] Sirota L, Punsky I, Bessler H. Effect of indomethacin on IL-1beta, IL-6 and TNFalpha production by mononuclear cells of preterm newborns and adults. Acta Paediatr. 2000;89: 331–335. 10772282

[89] Yanowitz TD, Yao AC, Werner JC, Pettigrew KD, Oh W, Stonestreet BS. Effects of prophylactic low-dose indomethacin on hemodynamics in very low birth weight infants. J Pediatr. 1998;132: 28–34. 10.1016/s0022-3476(98)70480-9 9469996

[90] Ng PY, Ireland DJ, Keelan JA. Drugs to block cytokine signaling for the prevention and treatment of inflammation-induced preterm birth. Front Immunol. 2015;6: 166 10.3389/fimmu.2015.00166 25941525 PMC4403506

